# Cilia in the Striatum Mediate Timing-Dependent Functions

**DOI:** 10.1007/s12035-022-03095-9

**Published:** 2022-11-02

**Authors:** Wedad Alhassen, Sammy Alhassen, Jiaqi Chen, Roudabeh Vakil Monfared, Amal Alachkar

**Affiliations:** 1 Department of Pharmaceutical Sciences, School of Pharmacy and Pharmaceutical Sciences, University of California-Irvine, 356A Med Surge II, Irvine, CA 92697-4625 USA; 2 UC Irvine Center for the Neurobiology of Learning and Memory, University of California-Irvine, Irvine, CA 92697 USA; 3 Institute for Genomics and Bioinformatics, School of Information and Computer Sciences, University of California-Irvine, Irvine, CA 92697 USA

**Keywords:** Cilia, Striatum, Timing, Behaviors

## Abstract

Almost all brain cells contain cilia, antennae-like microtubule-based organelles. Yet, the significance of cilia, once considered vestigial organelles, in the higher-order brain functions is unknown. Cilia act as a hub that senses and transduces environmental sensory stimuli to generate an appropriate cellular response. Similarly, the striatum, a brain structure enriched in cilia, functions as a hub that receives and integrates various types of environmental information to drive appropriate motor response. To understand cilia’s role in the striatum functions, we used loxP/Cre technology to ablate cilia from the dorsal striatum of male mice and monitored the behavioral consequences. Our results revealed an essential role for striatal cilia in the acquisition and brief storage of information, including learning new motor skills, but not in long-term consolidation of information or maintaining habitual/learned motor skills. A fundamental aspect of all disrupted functions was the “time perception/judgment deficit.” Furthermore, the observed behavioral deficits form a cluster pertaining to clinical manifestations overlapping across psychiatric disorders that involve the striatum functions and are known to exhibit timing deficits. Thus, striatal cilia may act as a calibrator of the timing functions of the basal ganglia-cortical circuit by maintaining proper timing perception. Our findings suggest that dysfunctional cilia may contribute to the pathophysiology of neuro-psychiatric disorders, as related to deficits in timing perception.

**Supplementary Information:**

The online version contains supplementary material available at 10.1007/s12035-022-03095-9.

## Introduction

Primary cilia, antennae-like microtubule-based organelles emanating from the cell surface, function as a signaling hub that senses environmental sensory stimuli and transduces them to generate appropriate cellular responses [1–4]. As an essential center for non-synaptic neuronal communications, cilia signaling is achieved through specific receptors and downstream pathways’ components such as the Sonic Hedgehog (Shh) signaling pathway, G-protein-coupled receptors (GPCRs), and ion channels [5–12].

Cilia’s dynamic structure, reflected by the vibrant length, morphology, and protein composition, allows cilia to quickly respond to environmental stimuli such as light, mechanical stimuli, pH, and chemical signals (signaling molecules, neurotransmitters, and nutrients) [13–19]. Although most ciliopathies are associated with cognitive impairments, cilia have only been scarcely investigated for their roles in higher-order cognitive functions [20–25]. We recently showed dysregulations of genes associated with cilia’s structural and functional components in four psychiatric disorders: schizophrenia, autism, bipolar disorder, and major depressive disorder [26]. Furthermore, many dysregulated cilia genes overlapped across these psychiatric disorders, indicating that common cilia signaling pathways’ dysfunctions may underlie some pathophysiological aspects of these psychiatric disorders.

Like cilia, though at larger spatial and organizational scales, the striatum, which comprises the primary input station to the basal ganglia, functions as a hub receiving and integrating various environmental information, including contextual, motor, and reward [27–31]. Striatal neurons process the information and project to the output structures of the basal ganglia, substantia nigra pars reticulata (SNr)/medial globus pallidus (GPm) (for review [32–35]). The striatum is enriched in cilia [36, 37]. Furthermore, a number of cilia-associated GPCRs are expressed in the striatum (e.g., dopamine receptors, serotonin receptor 6 (5-HT6), melanin-concentrating hormone receptor 1 (MCHR1), and the orphan receptors GPR88) [5–10, 38–56].

As a central part of cortico-basal ganglia-thalamic-cortico circuits, the striatum controls various executive functions, including motor movements, planning, decision-making, working memory, and attention [57–60]. Dysfunctions of cortico-basal ganglia-thalamic circuits are involved in several neurological and psychiatric (neuro-psychiatric) disorders such as attention-deficit hyperactivity disorder (ADHD), Huntington’s disease (HD), Parkinson’s disease (PD), schizophrenia (SCZ), autism spectrum disorder (ASD), Tourette syndrome (TS), and obsessive–compulsive disorder (OCD) [61–76]. The overlap in clinical features of these disorders suggests common signaling pathways that may underlie some pathophysiological aspects of these neuro-psychiatric disorders. Hence, it is tempting to ask whether cilia in the striatum mediate some of its functions and whether cilia are involved in psychiatric disorders associated with striatum dysfunctions.

According to our recent study, the striatum was the only brain structure that shared rhythmic cilia genes with every other brain region studied [18]. The spatiotemporal expressions of circadian cilia genes in the basal ganglia-cortical neurons follow the same sequential order of this circuitry in controlling movement, though on different time scales. Therefore, this study aims to examine the behavioral consequences of cilia ablation in the striatum and explore whether abnormal striatal cilia are a unifying pathophysiological factor in striatum-associated neuropsychiatric disorders. For this purpose, we used the loxP/Cre conditional deletion system to selectively delete the intraflagellar transport (IFT88) gene, an essential factor for primary cilia formation, from the dorsal striatum. We then monitored the behavioral phenotypes resulting from striatal cilia ablation, focusing on neuropsychiatric phenotypes/manifestations of the striatum functional domains.

## Material and Methods

### Animals

**Fig. 1:**
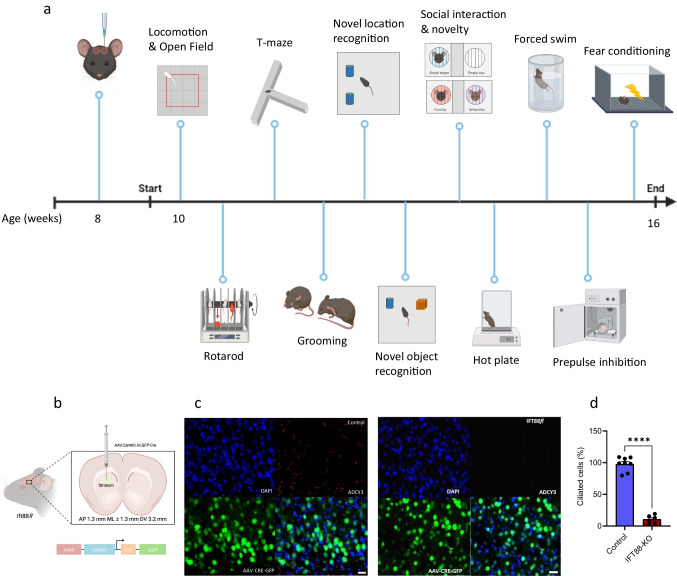

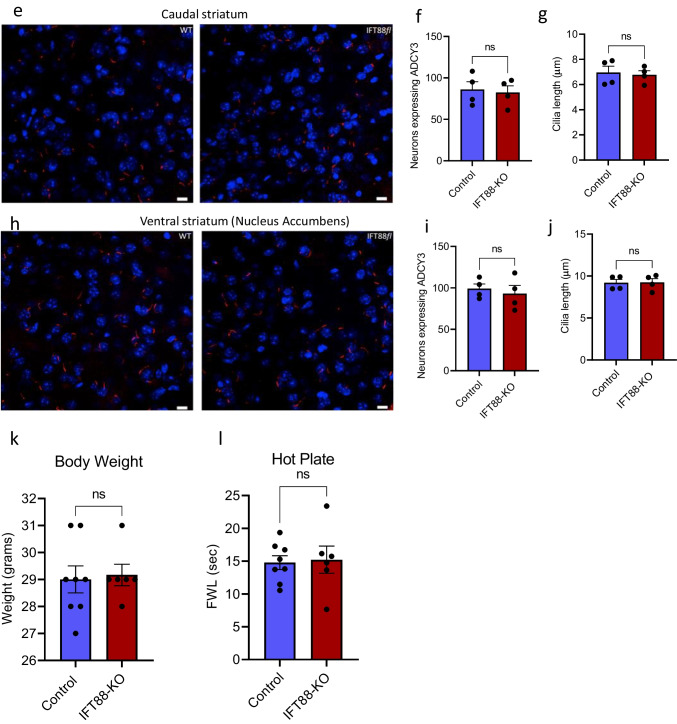
Selective cilia deletion in the striatum and confirmation of mice’s normal gross growth and well-being. **a** Schematic view of experimental design and behavior assays performed and their sequence. Diagram was created with the BioRender.com webpage. **b** Schematic showing bilateral viral injection into the dorsal striatum. **c** and **d** Verification of cilia removal in ciliated neurons of the striatum using immunostaining of ADCY3. Scale bar = 10 μm. **c** Representative images of ADCY3 immunostaining showing the intact cilia in the control mice and the conditional ablation of cilia in the dorsal striatum neurons of *Ift88*^*fl*^ mice (counterstained with DAPI, blue); **d** Quantification of the ciliated cells in the rostral-dorsal striatum (*n* = 8 control, 6 IFT88-KO). Unpaired *t*-test (*t* = 17.26, *P* < 0.0001) ****P < 0.0001. Data are presented as means ± S.E.M. Scale bar = 10 μm. **e–g** ADCY3 immunostaining in the caudal striatum. **e** Representative images of ADCY3 immunostaining in the caudal striatum showing that the selective removal of cilia from the dorsal rostral striatum does not affect **f** the number of ciliated cells (*t* = 0.30, *P* > 0.05) or **g** the cilia length (*t* = 0.30, *P* > 0.05, *n* = 4) in the caudal striatum. Scale bar = 10 μm. **h–j** ADCY3 immunostaining in the ventral striatum (nucleus accumbens). **h** Representative images of ADCY3 immunostaining in the ventral striatum showing that the selective removal of cilia from the rostral striatum does not affect **i** the number of ciliated neurons (*t* = 0.52, *P* > 0.05) or **j** the cilia length (*t* = 0.08, *P* > 0.05, *n* = 4) in the ventral striatum. Scale bar = 10 μm. **k** Effect of cilia removal on body weight to confirm normal gross growth (*n* = 8 control, 6 IFT88-KO). Unpaired *t*-test (*t* = 0.2463, *P* = 0.8096) revealed no significant difference in body weight. ns, not significant. Data are presented as means ± S.E.M. **l** Verification of well-being (*n* = 8 control, 6 IFT88-KO). Unpaired *t*-test (*t* = 0.2060, *P* = 0.8403) showed normal response to nociceptive stimulus. ns, not significant. Data are presented as means ± S.E.M

The *Ift88*^*fl*^ mice possess *loxP* sites flanking exons 4–6 of the intraflagellar transport 88 (*Ift88*) gene (Jackson Laboratories, #022,409). All experimental procedures were approved by the Institutional Animal Care and Use Committee of the University of California, Irvine, and were performed in compliance with national and institutional guidelines for the care and use of laboratory animals. The experimental design is illustrated in Fig. 1a. Only male mice were used in this study.

### Genotyping

The genotyping protocol was provided by Jackson Laboratory (JAX). DNA was extracted from mice using the following protocol. One millimeter of the tip of the tail was cut and digested in lysis buffer along with proteinase K overnight followed by isopropanol to precipitate the DNA, and ethanol to wash the pellet, and finally dissolved in TE buffer. DNA concentration and purity were checked. The DNA was used for the following PCR reaction. The following primers were used Forward 5′-GACCACCTTTTTAGCCTCCTG, Reverse 5′-AGGGAAGGGACTTAGGAATGA. Amplification was performed using KAPA2GFast HotStart PCR kit (Roche Cat. No 07960930001). Touchdown cycling was performed and ran on a 1% agarose gel with the homozygous at ~ 410 bp, heterozygous at 365 bp and ~ 410 bp, and wildtype at 365 bp.

### Stereotaxic Surgery

Eight-week-old male *Ift88*^*fl*^ mice were subjected to stereotaxic surgery along with wild-type littermates. Mice were anesthetized with 2% isoflurane and mounted on the stereotaxic frame. The skin between the eyes and ears was shaved, and an incision was made to uncover the skull and reveal bregma. A small hole was drilled bilaterally, and all mice received 0.5 μl of the adenovirus expressing Cre: AAV.CamKII.HI.GFP-Cre.WPRE.SV40 (titer 2.4 × 10^^13^ G.C./ml, serotype AAV9), which was bilaterally injected into the dorsal segment of the rostral striatum, at the stereotaxic coordinates anteroposterior at 1.3 mm, mediolateral at ± 1.3 mm, and dorsoventral at 3.2 mm (Fig. 1b). AAV.CamKII.HI.GFP-Cre.WPRE.SV40 was a gift from James M. Wilson (Addgene viral prep # 105,551-AAV9 http://n2t.net/addgene:105551; RRID:Addgene_105551). The skin was sutured with silk non-absorbable sutures, and mice were allowed a week to recover before behavioral experiments. After the last behavioral experiment at approximately 16 weeks old, mice were perfused, and AAV infection was analyzed.

### Behavioral Experiments

Two weeks after the surgery, mice were tested in a battery of behavioral paradigms in the following order (Fig. 1a): locomotion and stereotypy/open field, rotarod, grooming, social interaction and novelty, spontaneous T-maze alternation, novel object/location recognition, prepulse inhibition, forced swim, hot plate, and contextual fear conditioning. The sequence of specific assays spaced by 3–6 days inter-assay interval was adapted from previously published reports [77–79]. 

### Locomotor Activity Test and Open Field

The locomotor activity and open field assays were carried out as we previously described [78]. The experiment is divided into two phases of a total of 90 min experiment, which is divided into 30 min acclimation and 60 min locomotor activity test [78]. Animals were placed in a 40 × 40 cm locomotion chamber (Med Associates, Inc.), and their activity was logged every 5 min, over the assay duration using Activity Monitor 5 software (Med Associates, Inc.).

Open field assay was carried out in the first 10 min after placing the mice into the chambers, and the distance traveled and activity time in the central and peripheral zones were recorded and analyzed using Activity Monitor 5 software.

### Rotarod Test

In order to evaluate motor coordination, mice were subjected to the rotarod assay. Animals were placed on an elevated rotating rod divided into 5 lanes with trip plates below each lane to stop the timer when a subject falls. Each trial is 420 s with a starting speed at 4 RPM that continues to incrementally speed up to 60 RPM. Animals were subjected to 3 trials with 15 min between each trial. Data were analyzed based on latency and speed to fall over each trial.

### T-maze Spontaneous Alternation

Mice were placed in the entrance at the base of the T-maze. Mice were acclimated at the base of the T-maze for 30 sec. After acclimation, the doors were opened, and animals were free to explore either the left or right arm of the maze. After a choice had been made, the door was closed, allowing the animal to explore the sidearm chosen for 30 sec. Mice were then returned to the maze base to start the subsequent trial. Eight total trials were carried out with 7 possible alternations, and the alternation percentage was calculated as 100x (number of alternations/7). The time taken to make the alternation decision was recorded as well.

### Self-Grooming Behavioral Assay

Mice were placed in an empty cage for 20 min. They were first allowed to acclimate to the environment for 10 min and then were monitored for grooming activity for the last 10 min. Time spent grooming over the 10 min was recorded. Videos were recorded, and grooming was manually scored for grooming activities such as licking, face swiping, scratching, or nibbling. Each video was scored by three persons blinded to the animal genotype, and the average of the scores was recorded.

### Social Interaction and Social Novelty

The 3-chamber box used for these assays is a rectangular plexiglass box consisting of a left, middle, and right chamber with removable doors, which separate the chambers. Empty mesh wire cups were placed in the middle of both the left and right chambers. In the social interaction assay, mice were allotted 5 min to explore the middle chamber. After 5 min, a control mouse of the same gender, age, and strain as the experimental mouse was placed inside one of the cups in either the right or left chamber. The doors were then removed, allowing the experimental mice to explore all three chambers for 10 min. The total time experimental mice spent interacting with both the empty cup and the control mouse cup was recorded.

Immediately following the social interaction assay, the social novelty assay began. The experimental mouse was returned to the middle chamber, and a new control mouse was placed underneath the empty cup. Doors were removed, and the experimental mouse was allotted 10 min to explore all three chambers. The total time experimental mice spent interacting with the mouse from the social interaction assay and the novel mouse was recorded. ANY-maze software (Stoelting, Wood Dale, IL, USA) was used to record and analyze these interactions.

### Novel Object Recognition

Novel object recognition (NOR) consists of a training and testing phase. All mice were handled for 1–2 min a day for 3 days prior to the training phase. Mice were then allowed to habituate to the experimental apparatus, a rectangular box for 3 consecutive days without the presence of objects. During the training phase, mice were exposed to two identical objects in the apparatus and allowed to explore for 10 min. After 24 h, mice were subjected to the testing phase, where they were allotted 5 min to explore the apparatus with the familiar object and a novel object. The total time each subject mouse spent interacting with both the familiar and novel object was recorded individually. ANY-maze software (Stoelting, Wood Dale, IL, USA) was used to document and analyze these interactions.

### Novel Location Recognition

Novel location recognition (NLR) consists of the training and testing phase. All mice were handled for 1–2 min a day for 3 days prior to the training phase. Mice were then allowed to habituate to the experimental apparatus, a rectangular box for 3 consecutive days without the presence of objects. During the training phase, mice were exposed to two identical objects in the apparatus and allowed to explore for 10 min. After 24 h, mice were subjected to the testing phase, where one location of the object is moved. Mice were allotted 5 min to explore the apparatus with the familiar location and a novel location. The total time each subject mouse spent interacting with both the familiar and novel location was recorded individually. ANY-maze software (Stoelting, Wood Dale, IL, USA) was used to document and analyze these interactions.

### Forced Swim

Mice were placed in a cylinder containing water at 25 °C for 6 min. Mice were recorded, and following the assay, the last 4 min were scored and analyzed. The immobility time or time the mice spent floating was recorded. ANY-maze software was used to record and analyze immobility time (Stoelting Co.).

### Prepulse Inhibition

Mice were habituated to the startle chambers for 5 min with 65 dB of background noise. The PPI sessions consisted of 5 trials, either no stimulus (65 dB), 3 prepulse (20-ms prepulse at 68 dB, 71 dB, or 77 dB, a 100-ms interstimulus interval, followed by a 40-ms duration startle stimulus at 120 dB. The amount of prepulse inhibition was calculated as a percentage score for each acoustic prepulse intensity: % PPI = 100 - ([(startle response for prepulse + pulse trials) / (startle response for pulse − alone trials)] X 100).

### Hot Plate

Mice were habituated to the hot plate with no heat to establish a baseline. Following the habituation, mice were subjected to a 52 °C hot plate, and were monitored for an initial response of either a rear paw lift, paw shake, or paw lick in response to the heat or once the cutoff time is reached. Once the initial response was seen, the time was recorded, and animals were returned to their home cage.

### Contextual Fear Conditioning

This assay consists of a training and a testing session 24 h following the training session. On day 1, mice were placed in the conditioning chamber for 3 min, received a 2-s 0.7 mA foot shock at 2.5 min, and were placed back into their home cage on day 2, animals were returned to the same chamber for 5 min without shock. Freezing behavior was measured pre- and post-shock sessions and was scored as freezing (1) or not (0) within a 5-s interval and calculated as 100x (the number of intervals of freezing / total intervals).

### Immunohistochemistry

Ninety minutes after the fear conditioning assay, mice were anesthetized with isoflurane and transcardially perfused with saline and 4% paraformaldehyde (PFA). Brains were harvested, kept in PFA overnight, and switched to 30% sucrose. Brains were coronally sectioned at 20 μm using a microtome. Using the Allen Brain Atlas, 3–4 sections were selected from specific regions of interest. Sections were blocked with goat serum in PBS with 0.3% Triton X-100 for 1 h. Next, brain sections were incubated in blocking buffer with the primary antibody, either cFos, 1:500 (Abcam cFos ab190289 Rb pAb to cFos Lot#: GR339395) or ADCY3, 1:500 (LSBIO-C204505 ADCY3 Lot#: 193037). Following the primary antibody incubation, sections were washed with PBS and then incubated with the secondary antibody, 1:500 (Invitrogen AlexaFluor546 goat anti-rabbit ref: A101035 Lot: 2,273,730), and DAPI, 1:10,000 (Thermo Scientific Ref: 62,248 Lot: WF3296471). Sections were washed with PBS and mounted on slides. Images were carried out using the Leica SP8 confocal microscope with a 63 × objective lens (UCI optical biology core facility) for visualizing cilia, and Keyence BZ-9000 microscope with a 10x objective lens for visualizing cFos positive neurons. 

### Image Analysis

cFos positive neurons and ADCY3 were counted bilaterally, and the mean of the three sections per 4–6 brains was calculated. All image analysis and cell counts were performed in Fiji (ImageJ). Automatic particle quantification and analysis methods were used for counting cFos labeled neurons. Briefly, images were opened in Fiji, and color channels were split into the appropriate color. Color images were then converted to grayscale. All regions were defined with specific dimensions across all images for cohesiveness. Once in grayscale, a threshold was set to highlight fluorescent particles and create a binary image. The "analyze particle" function was used to select the size of particles and set the circularity of the particles. Cilia length was measured using the line measurement tools in Fiji, and the length unit was converted from pixels to micron.

### Statistical Analysis

GraphPad Prism (GraphPad Software, Inc.) was employed to perform statistical analysis. Data were presented as means ± S.E.M. Results were analyzed using student *t*-test or ANOVA followed by the appropriate post hoc comparisons, and *P* < 0.05 was considered statistically significant.

## Results

### Selective Deletion of Cilia in the Striatum

To examine the physiological role of primary cilia in the striatum, we used the conditional deletion system and disrupted intraflagellar transport (IFT) machinery by deleting the Ift88 gene. Stereotactic infusion of IFT88fl/fl mice with AAV.CamKII.HI.GFP-Cre into the striatum (Bregma level 1.3 mm, ± 1.3 mm, 3.2 mm) resulted in mice with primary cilia deficiency exclusively in the dorsal part of the rostral striatum (Fig. 1b).

Cilia deletion from the dorsal-rostral striatum did not affect the number of striatal neurons, indicating that the dorsal-rostral striatum of IFT88-KO mice is intact, and the neurons were viable. Next, we performed a coexpression analysis of primary cilia with GFP-Cre via immunohistochemistry, using an antibody against ADCY3, a well-known marker of primary cilia. While the numbers of neurons expressing primary cilia and the cilia length were comparable in caudal striatum and nucleus accumbens between the control and IFT88-KO mice, these numbers were markedly reduced in the dorsal-rostral striatum of IFT88-KO mice (Fig. 1c–j). Furthermore, comparable body weights and normal response to nociceptive stimulus (hot plate) confirmed that the IFT88-KO mice have normal gross growth and well-being (Fig. 1k, l).

### Primary Cilia in the Striatum Are Required to Maintain Normal Motor Coordination But Not the Spontaneous Motor Activity

**Fig. 2:**
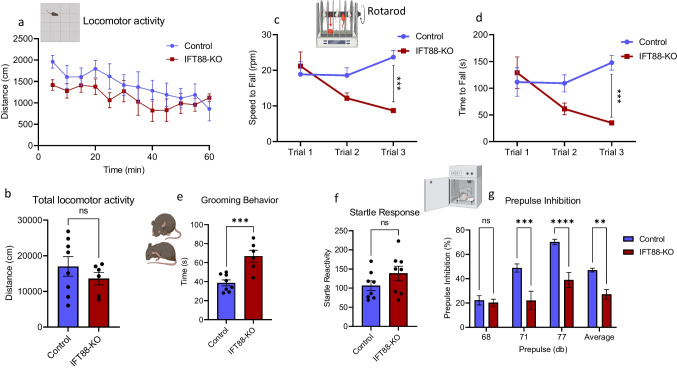
Primary cilia ablation in the striatum affects motor and sensorimotor-related behaviors. **a** Distance traveled in the last 60 min of the locomotor assay (*n* = 8 control, 6 IFT88-KO). Two-way ANOVA (F_(11, 144)_ = 0.4143, *P* > 0.05) followed by Bonferroni’s post hoc test showed that IFT88-KO mice displayed similar locomotor activity to the control mice; ns, not significant. **b** Total locomotor activity in the locomotor assay. Unpaired *t*-test (*t* = 0.9608, *P* = 0.3556), ns, not significant. Data are presented as means ± S.E.M. **c** and **d** Motor skill learning on the accelerated rotarod. **c** Speed to fall in rotarod assay (*n* = 8). Two-way ANOVA (cilia removal factor: F_(1,42)_ = 9.399, *P* = 0.0038, trial factor F_(2,42)_ = 1.927, *P* > 0.05) followed by Bonferroni post hoc test: IFT88-KO vs control, **P < 0.001. Data are presented as means ± S.E.M; **d** Latency to fall in rotarod assay (*n* = 8). Two-way ANOVA (cilia removal factor: F_(1,42)_ = 9.246, *P* = 0.0041, trial factor: F_(2,42)_ = 1.93, *P* > 0.05) followed by Bonferroni post hoc test: IFT88-KO vs control, ***P < 0.001. Data are presented as means ± S.E.M. **e** Repetitive behavior in grooming behavior assays (*n* = 8 control, 6 IFT88-KO), unpaired *t*-test (*t* = 4.357, *P* = 0.0009), IFT88-KO vs control. Data are presented as means ± S.E.M. **f** and **g** Performance of mice in PPI assay. **f** Startle reactivity in prepulse inhibition assay (*n* = 8), unpaired *t*-test (*t* = 1.424, *P* = 0.1763), ns, not significant. **g** Prepulse inhibition in PPI assay (*n* = 8), two-way ANOVA (cilia removal factor: F_(1,56)_ = 41.69, *P* < 0.0001, prepulse intensity factor: F_(3,56)_ = 19.55, *P* < 0.0001), control vs IFT88-KO, **P < 0.01, ***P < 0.001, ****P < 0.0001. Data are presented as means ± S.E.M

Although the striatum is identified as a brain site for motor control timing, it is unknown whether striatum primary cilia play a role in regulating motor functions. To address this question, spontaneous motor activity and motor coordination were monitored in IFT88-KO mice. After the habituation period, IFT88-KO mice displayed similar locomotor activity to the control mice, measured by distance traveled (Fig. 2a, b). However, the locomotor activity in the first 10 min after placing mice into the open box was significantly lower in the IFT88-KO than in the control mice. In addition, IFT88-KO mice showed a reduced latency to fall and a lower rotation speed at which they fall in the rotarod test (Fig. 2c, d), indicating an impairment of motor coordination in the IFT88-KO mice. This may also be interpreted as impairment of procedural learning as trial 1 shows IFT88-KO mice just as coordinated as the control mice.

### Ablation of Primary Cilia in the Striatum Increases Repetitive Behavior and Impairs Sensorimotor Gating

The IFT88-KO mice showed a higher level of compulsive grooming than the control mice (Fig. 2e), indicating an enhanced compulsive, repetitive behavior in these animals. Grooming was measured as the time mice spent face swiping, paw licking, rubbing of the head and ears, and cleaning the entire body. IFT88-KO mice also exhibited reduced values of prepulse inhibition of the startle reaction at 71 dB and 77 dB prepulses, as well as the average prepulse, although the startle reactions were comparable to those in the control mice (Fig. 2f, g). The average PPI value was also lower in IFT88-KO mice than the control ones. These results indicate that ablation of cilia in the striatum causes a deficit in sensorimotor gating without affecting the startle reaction.

### Primary Cilia in the Striatum Are Not Involved in Anxiety, Sociability, and Depressive-Like Behaviors

**Fig. 3:**
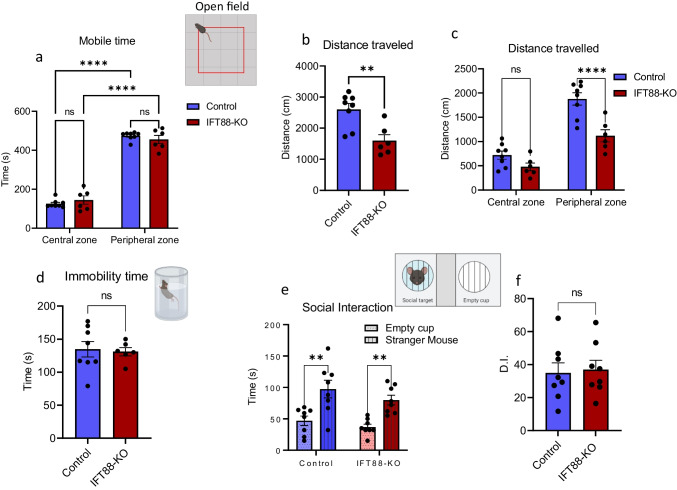
Primary cilia removal in the dorsal striatum does not affect anxiety, sociability, and depressive-like behaviors. **a** Time spent in central vs time in peripheral zones in open field assay (*n* = 8 control, 6 IFT88-KO), two-way ANOVA (cilia removal factor: F_(1,24)_ = 0.00, *P* > 0.999, zone factor: F_(1,24)_ = 538.1, *P* < 0.0001) followed by Bonferroni post hoc test. ****P < 0.0001. Data are presented as means ± S.E.M. **b** Total distance traveled in open field assay, unpaired *t*-test (*t* = 3.598, *P* = 0.0037), IFT88-KO vs control, **P < 0.01. Data are presented as means ± S.E.M. **c** Distance traveled in central vs time in peripheral zones in open field assay (*n* = 8 control, 6 IFT88-KO), two-way ANOVA (cilia removal factor: F_(1,24)_ = 21.1, *P* = 0.0001, zone factor: F_(1,24)_ = 68.31, *P* < 0.0001), followed by Bonferroni post hoc test, ****P < 0.0001. **e** Social interaction (*n* = 8), two-way ANOVA (cup factor: F_(1,28)_ = 26.25, *P* < 0.0001, cilia removal factor: F_(1,28)_ = 2.28, *P* = 0.1420); Empty cup vs stranger mouse, **P < 0.0001. Data are presented as means ± S.E.M. **f** Social interaction discrimination index, unpaired *t*-test (*t* = 0.2422, *P* = 0.8121), control vs IFT88-KO, ns, not significant. Data are presented as means ± S.E.M

To test whether cilia ablation affects anxiety-like behavior, we used open field assay, which is based on the assumption that mice placed in a new environment tend to avoid open space and, therefore, spend more time in the peripheral than central arenas. Cilia ablation did not affect anxiety-like behavior, revealed by the longer times spent in the peripheral than central arenas in IFT88-KO mice, which were comparable to those in the control mice (Fig. 3a). Although the total distance and the distance traveled in the peripheral zone were significantly lower in the IFT88-KO mice than the control mice (Fig. 3b, c), this does not indicate an altered anxiety-related behavior; rather, it reflects a slower response to the new environment. The immobility time in the forced swim test, which measures helplessness behavior, was similar in the control and IFT88-KO mice (Fig. 3d). Furthermore, IFT88-KO mice showed normal social behavior, revealed by spending significantly more time with a stranger mouse than an empty cup (Fig. 3e, f). These results indicate that cilia ablation in the striatum does not affect anxiety, sociability, and depressive-like behavior.

### Primary Cilia in the Striatum Are Required for Spatial Working Memory But Not Other Memory Types

We examined the effects of cilia ablation in the striatum on different types of memories, including spatial working memory, social recognition memory, object recognition memory, spatial memory (location recognition), and contextual memory. Spatial working memory was tested in the T-maze paradigm, which is based on the mice’ tendency to repeatedly alternate between the right and left arms in order to optimize their navigation of their environment [80]. The short-term social memory (social recognition) was tested using the three-chamber assay, which is based on the instinctive tendency of mice to investigate and spend more time with unfamiliar social subjects (strange mouse) than familiar ones [81]. Long-term memory formation involves encoding, short-term memory consolidation (storage), and long-term memory reconsolidation and retrieval [82] and is usually tested in mice 24 h after conditioning [71].

**Fig. 4:**
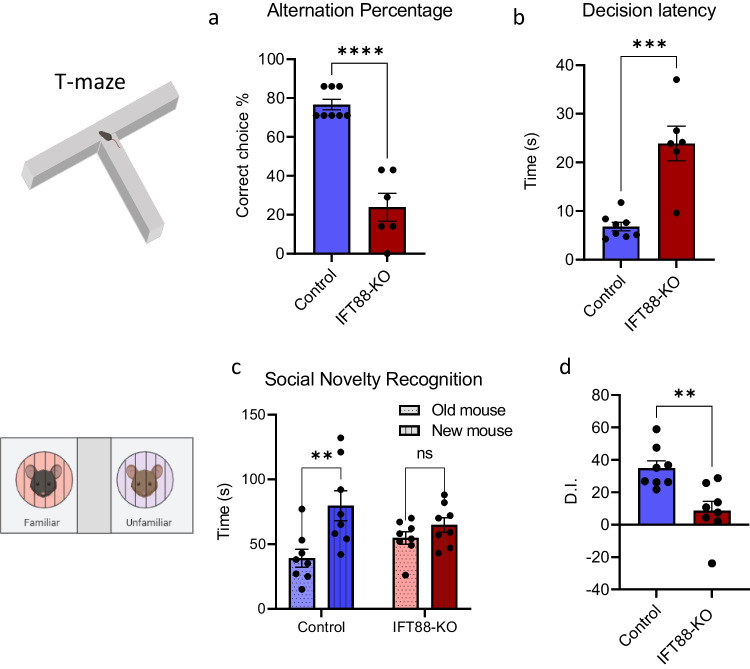

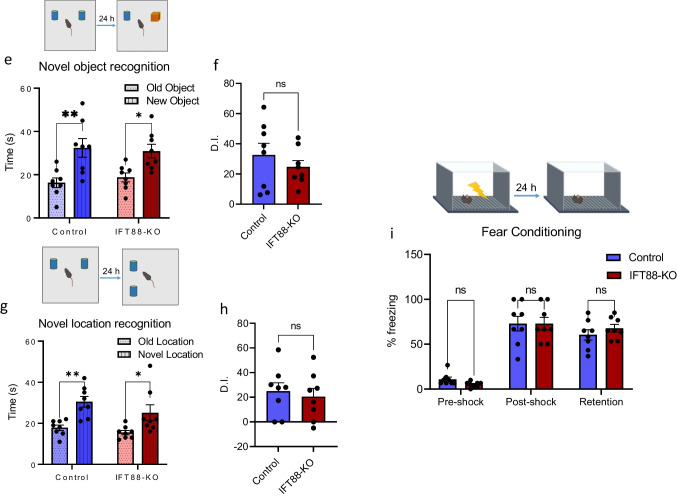
Primary cilia in the striatum are required for spatial working memory but not other memories. **a** Spatial working memory alteration percentage in T-maze (*n* = 8 control, 6 IFT88-KO), unpaired *t*-test (*t* = 7.679, *P* < 0.0001), control vs IFT88-KO, ****P < 0.0001, **b** Decision latency in T-maze test (*n* = 8 control, 6 IFT88-KO), unpaired *t*-test (*t* = 5.295, *P* = 0.0002), control vs IFT88-KO, ***P < 0.001, **c** social novelty recognition (*n* = 8), two-way ANOVA (cilia removal factor: F_(1,28)_ = 10.90, *P* = 0.0026, novel mouse factor: (F_(1,28)_, *P* = 0.9612), followed by Bonferroni post hoc test: old mouse vs new mouse, **P < 001, ns, not significant. Data are presented as means ± S.E.M. **d** Discrimination index in social novelty recognition (*n* = 8), unpaired *t*-test (*t* = 3.604, *P* = 0.0029), control vs IFT88-KO, **P < 0.01. **e** Novel object recognition (*n* = 8), two-way ANOVA (object factor: F_(1,28)_ = 21.24, *P* < 0.0001, cilia removal factor: F_(1,28)_ = 0.02662, *P* = 0.8716): old object vs new object: *P < 0.05, **P < 0.01. Data are presented as means ± S.E.M. **f** Discrimination index in novel object recognition (*n* = 8), unpaired *t*-test (*t* = 0.90, *P* = 0.38), control vs IFT88-KO, ns, not significant. Data are presented as means ± S.E.M. **g** Novel location recognition (*n* = 8), two-way ANOVA (object factor: F_(1,28)_ = 20.30, *P* < 0.0001, cilia removal factor: F_(1,28)_ = 0.2.46, *P* = 0.8716): *P < 0.05, **P < 0.01. **h** Discrimination index in novel location recognition (*n* = 8), unpaired *t*-test (*t* = 0.46, *P* = 0.65), control vs IFT88-KO, ns, not significant. **i** Fear conditioning (*n* = 8), two-way ANOVA (*P* > 0.05) followed by Bonferroni post hoc test: control vs. IFT88-KO. ns, not significant. Data are presented as means ± S.E.M

The spatial working memory was significantly impaired in the IFT88-KO mice, revealed by the higher incorrect choices in these mice than in the control mice (Fig. 4a). Associated with working memory impairment in IFT88-KO, the latency for decision-making was significantly longer in these mice compared with than the control mice (Fig. 4b). Interestingly, while the ablation of primary cilia in the striatum did not affect social behavior, it caused an impairment in social recognition memory (Fig. 4c, d), as reflected by the similar time IFT88-KO mice spent with the old and new stranger mice.

Ablation of primary cilia in the striatum did not affect the object recognition memory, as evidenced by the more time mice spent with the novel object than the old object (Fig. 4e, f). Similarly, in the novel location recognition assay, IFT88-KO spent more time with the novel location than the old location (Fig. 4g, h), indicating a normal spatial memory. In addition, the contextual memory, measured using the fear conditioning test, was intact in the IFT88-KO mice, as revealed by the similar freezing time on the test day compared with the control mice (Fig. 4i).

### Neural Activity Is Changed in Striatal Input and Output Nuclei Pathways

**Fig. 5:**
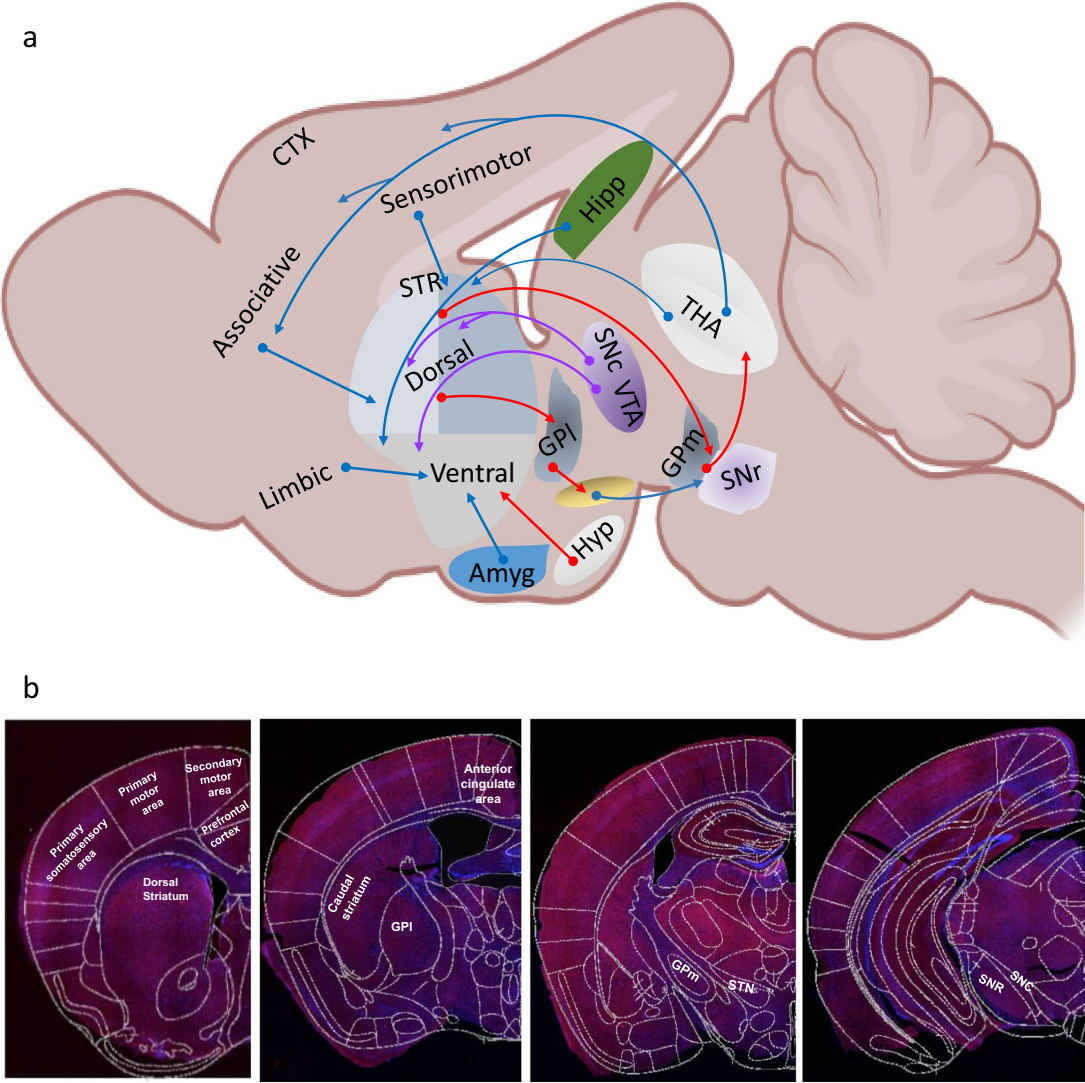

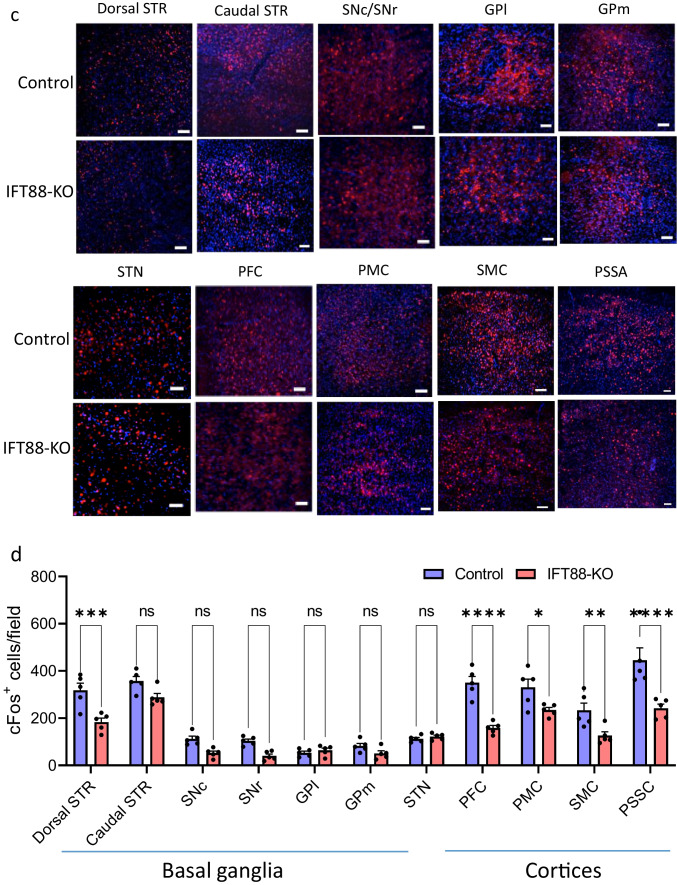
Effects of cilia removal in the dorsal striatum on cFos expression in the striatum, its input and output structures. **a** Schematic view of neuronal circuits in mice brain. Amy, amygdala; CTX, cortex; GPL, lateral globus pallidus; GPm, medial globus pallidus; Hipp, hippocampus; Hyp, hypothalamus; SNc, substantia nigra pars compacta; SNr, substantia nigra pars reticulata; STN, subthalamic nucleus; STR, striatum; THA, thalamus; VTN, ventral tegmental area. Diagram was created with the BioRender.com webpage. **b** cFos immunostaining in four levels of the mouse brain. **c** Representative images of cFos immunostaining in the striatum and its input and output structures: striatum (STR), substantia nigra pars compacta (SNc), substantia nigra pars reticulata (SNr), lateral globus pallidus (GPl), medial globus pallidus (GPm), subthalamic nucleus (STN), prefrontal cortex (PFC), primary motor cortex (PMC), secondary motor cortex (SMC), primary somatosensory area (PSSA). Scale bar = 10 μm. **d** Quantification of the cFos-positive cells in the control and IFT88-KO mice. Two-way ANOVA, control vs IFT88-KO (F_(1,88)_ = 94.28, *P* < 0.0001), *P < 0.05, **P < 0.01, ***P < 0.001, ****P < 0.0001, ns, not significant. Data are presented as means ± S.E.M; *n* = 3 sections of 5 mice per group

The expression of the immediate-early gene cFos was used as a molecular marker of neural activity. We examined cFos immunoreactivity (number of cFos-positive cells) in structures that are parts of striatal circuits and those known to project to or receive projections from the striatum (Fig. 5a, b). First, the rostral dorsal striatum, but not the caudal striatum of IFT88-KO mice, exhibited a significant decrease of cFos immunoreactivity (Fig. 5c, d). Within the basal ganglia circuit, there was a trend for cFos immunoreactivity reductions in the output regions (SNr and the GPm), but not in the nuclei of the indirect pathway structures (lateral globus pallidus and subthalamic nucleus) (Fig. 5c, d). The main input regions to the striatum include the dopaminergic neurons of the substantia nigra pars compact (SNc) and the glutamatergic neurons of the cortices. While IFT88-KO mice exhibited significant decreases in cFos immunoreactivity in several cortices, including the prefrontal cortex, primary motor area, secondary motor area, and somatosensory area, there was a trend for cFos decrease in the SNc, albeit not significant (Fig. 5c, d).

## Discussion

In this study, we examined the role of cilia in the striatum using a selective conditional deletion system. Our data provide the first evidence for the essential role of striatal primary cilia in specific functions of the striatum, namely, sensorimotor and executive functions.

### Methodological Considerations

To examine whether striatal cilia mediate some of the striatum functional domains, we used loxP/Cre technology to selectively delete IFT88, an essential protein for cilia genesis, from the dorsal striatum. The deletion of IFT88 resulted in cilia ablation in the dorsal striatum, as evidenced by the profound decrease in ADCY3-immunostaining. We monitored the behavioral phenotypes resulting from striatal cilia ablation, focusing on neuropsychiatric phenotypes related to three striatum functional domains: the sensorimotor, associative, and limbic domains. The sensorimotor domain is essential for habitual behaviors that are automatically evoked, whereas the associative (cognitive) domain is responsible for driving consciously formed actions and goal-directed behaviors. The dorsal striatum mediates these two functional domains, whereas the limbic domain, associated with motivational and affective behaviors, is sited in the ventral striatum (nucleus accumbens in rodents) [83–88].

### Cilia in the Dorsal Striatum Are Necessary for Sensorimotor Learning and Execution of Goal-Directed Behaviors, But Not Affective Behaviors

Cilia ablation from the dorsal striatum did not affect spontaneous motor in mice, evidenced by the normal locomotor activity in the IFT88-KO mice. However, motor activity was lower in IFT88-KO directly after placing them into the open field apparatus, indicating that their reactions to a new environment are diminished. Interestingly, the performance of cilia-ablated mice in the first trial of the rotarod assay was comparable to that of the control mice, indicating that cilia-ablated mice can walk normally on the rotarod. In contrast to the control group, however, in the second and third trials, cilia-ablated mice’ performance not only did not improve but also worsened. The proper performance in rotarod assay reflects motor coordination and learning, which are dependent on striatal function in processing and integrating new sensory information and coordinating the time sequence of motor response [89–92]. Our findings indicate an essential role for striatal cilia in acquiring new motor skills learning, but not in maintaining habitual or already learned motor skills. While we cannot make a concrete conclusion on whether this function of cilia is mediated through processing and integrating new sensory information or by coordinating the time sequence of motor response, we speculate that cilia might be involved in both mechanisms.

In support of this assumption, we found that cilia ablation in the striatum impaired sensorimotor gating, as revealed by the reduced PPI levels [93]. Sensorimotor gating, a largely striatum-thalamus-dependent phenomenon, represents the ability of the brain to respond to a sensory stimulus by suppressing a motor response. Through this mechanism, the brain filters irrelevant sensory information input, prior processing and transmitting to motor output systems. The PPI deficits in cilia-ablated mice further support an essential role for cilia in the sensorimotor integration process and motor executing actions.

The role of cilia in sensorimotor functions is further evidenced by our finding that cilia-ablated mice exhibited excessive repetitive behavior, revealed by increased self-grooming. Repetitive behaviors result from excessive automatized actions or exaggerations of habitual behaviors, for which the dorsal striatum plays an essential role [94–96]. Our results, thus, indicate that these actions of the dorsolateral striatum are mediated through its primary cilia. One important question posed by our findings is whether the failure of acquiring new motor skills in cilia-ablated mice is causally correlated with their enhanced repetitive behavior. Robust evidence, lending a premise to this posit, is that repetitive behaviors arise from the inflexibility in switching and transitioning between habitual and novel motor behavioral patterns [97–99]. Accordingly, cilia may provide an adaptation mechanism that adjusts the transition of habitual behaviors to repetitive behaviors.

We tested the effects of cilia ablation in the striatum on the three types of memory: working memory, short-term memory, and long-term memory. Our results reveal that striatal cilia ablation impairs spatial working memory and short-term social memory and delays decision and task execution in the working memory test but does not affect any long-term memory types.

Working memory encodes and stores information in its temporal context and permits its further manipulation to guide decision-making and behavior execution [100, 101]. The dorsal striatum is involved in the initial storage of temporal information in working memory, and dysfunctions of the striatum or dopaminergic projections to the striatum are known to impair the execution of working memory [102–104]. The striatum also plays an essential role in social recognition, in which different sensory cues are acquired for coding social information [105–107]. Taken together, our results indicate that cilia in the striatum are essential for initial acquisition and brief storage of information but not for its long-term consolidation or retrieval. Our results also suggest that cilia are key components of brain networks for action selection and timing of the decision process. Previous studies have shown that cilia removal from the hippocampus and cortex resulted in deficits in fear conditioning memory [23, 108]. However, our finding of the lack of an impact of cilia loss from the rostral striatum on the contextual fear memory does not seem to contradict with these previous results, since the hippocampus and cortex rather than the striatum are known to be involved in contextual fear memory formation.

### Dorsal Striatal Cilia Ablation Does Not Affect Limbic Functional Domains

Cilia ablation in the dorsal striatum did not impair motivational and affective functions, as revealed by the normal sociability, anxiety, nociception, and depressive-like behaviors in the cilia-ablated mice. These results are interesting, though not surprising, given that the dorsal striatum has a minor role in these functions compared with the ventral striatum (nucleus accumbens in rodents), which is the main site for limbic functional domains [109–111].

### Striatal Cilia Ablation Alters Neuronal Activities in Striatum Input Structures

Associated with the behavioral phenotype induced by striatal cilia ablation, cFos expression, as a marker for neural activity, was altered in the dorsal striatum itself and in the cortical structures that project to the dorsal striatum, including nuclei of sensorimotor and associative cortical-basal ganglia-thalamic-cortical circuits. The striatum receives projections from the cortex, thalamus, and SNc [28, 31, 112], and its neurons project to the outputs of the basal ganglia SNr/GPm via the direct and indirect pathways via GPl and the subthalamic nucleus (STN) (for review [32–35]). The primary motor cortex is responsible for generating the signals that control movement execution, whereas the secondary motor areas control motor planning. The thalamus, a main output target of the basal ganglia, is under the inhibitory (GABAergic) tone of the SNr and GPm, and in its turn projects, using glutamate transmitter, back to the cortex [113–116]. On the other hand, the dopaminergic neurons of the SNc project to the striatum, stimulating the direct pathway through D1 receptors and inhibiting the indirect pathway via D2 receptors. Surprisingly, none of the basal ganglia structures that receive projections from the striatum showed altered cFos expression. This finding is interesting and, together with the finding of decreased cFos in the cortical regions, indicates that cilia removal from the striatum decreases the activity of presynaptic neurons projecting to the striatum more than neurons that receive projections from the cilia-ablated neurons. It is noteworthy, however, that cFos expression was analyzed in mice' brains 90 min after the fear conditioning retrieval test. Given that cilia-ablated mice showed normal performance in this assay, the profound changes in cFos expressions in several brain regions suggest that striatal cilia ablation caused alteration in the constitutive cFos levels [117, 118].

### Striatal Cilia as a Modulator of Timing Perception

An interesting observation made of the reconciliation of our results is that the disrupted functions in striatum cilia-ablated mice share a common fundamental aspect: “impaired time perception,” i.e., losing the ability of quick and timely adjustment of behavior in response to changing environmental cues and, thus, failing to maintain appropriate, goal-directed motor response [119]. Time perception is embedded in the processing and integration of environmental sensory information and in motor and executive actions. Timing judgment allows for the timely selection of appropriate responses to the environment sensory. In this sense, prepulse inhibition of startle reaction, repetitive motor, and motor coordination all involve a sequence of motor actions, for which execution requires precise timing and coordination, usually in the millisecond timescale. Therefore, the exclusive impairments of these functions in cilia-ablated mice may indicate a disruption of time judgment and adjustment, which may impede the successful execution of these motor activities [120–125]. Time perception is also critical for cognitive processes. Successful performance of working memory, attention, decision-making, and executive function requires accurate and precise timing judgment, usually within a millisecond to minute timescale [126–133]. In the context of working memory, the encoding, maintenance, and synchronization of stimulus attributes are presented in specific temporal sequences [134, 135]. Memory formation begins when environmental stimuli first elicit a timing mechanism, in which information about elapsed time is stored in working memory or short term. When the stimulus ends or another event happens, the value of that duration is stored in long-term memory [100, 136]. The impairments of working memory and the delay in the decision and executive function in cilia-ablated mice further support the speculation of interval timing disruption in these mice. This speculation is in line with the known role of the basal ganglia circuit in performing central clock functions in the brain [137, 138]. Particularly, the SNc and striatum are the two basal ganglia regions necessary for interval timing and are parts of cortical-basal-ganglia circuits that form neuronal clocks [139–146]. These circuits coordinate the estimation and reproduction of time, wherein dopamine modulates the clock speed and timing judgment functions [147–149]. For example, increased dopamine levels result in a faster internal clock process, whereas decreased dopamine slows down the clock speed [147–149]. Furthermore, dysfunctions of the striatum have been shown to cause impairments of interval-timing judgments, particularly as related to the spatial working memory [104, 150–152]. Interestingly, we recently found that most cilia transcripts in the primate basal ganglia-cortical circuit are circadian, and they peak in a sequential pattern similar to the sequential order of activation of structures of this circuit during movement coordination albeit on completely different time scales. Taken together, our findings, in line with robust evidence from the literature, suggest a critical role for striatal cilia in the brain’s central clock function.

### Implications of Cilia Dysfunctions in Neuropsychiatric Disorders

Intriguingly, the distinctive behavioral phenotype induced by striatal cilia ablation in mice appears to pertain to clinical manifestations of specific neurological and psychiatric disorders related to both the striatum functions and timing deficits. We base our view on the following notions:

1. The behavioral deficits in cilia-ablated mice represent a cluster that extensively overlaps across specific disorders, including SCZ, PD, HD, ASD, OCD, ADHD, and TS, with some of these deficits more significant in particular disorders than in others. Motor coordination deficits, for example, are the main features of PD and HD but are also observed in TS, ASD, OCD, SCZ, and ADHD [153–162]. On the other hand, repetitive behavior is a common feature of ASD, TS, OCD, and SCZ but is also found in ADHD and PD [163–174]. While sensorimotor gating deficit is a characteristic feature of SCZ, it is also observed in ASD, HD, PD, ADHD, OCD, and TS [175–187]. Furthermore, social recognition memory is impaired in ASD, SCZ, and OCD [188–193]. Lastly, decision execution and working memory deficits are common features in SCZ, ASD, ADHD, PD, HD, and OCD [194–204].
2. Dysfunctions of the striatum, as a target of dopaminergic pathways and an essential part of the cortico-basal ganglia-thalamic circuits, underlie fully or partially the pathophysiology of these neuro-psychiatric disorders [61–76].
3. A common feature of striatum-related disorders and the seven discussed disorders is a profound decrease in patients’ ability to accurately calculate the timing of the initiation and termination of voluntary actions [205–220]. While time perception deficits have been extensively studied in the PD, SCZ, ADHD, and HD individuals, timing judgment deficits in ASD, OCD, and TS have recently begun to receive attention [205–220].

It is also important to note that it is possible that the disruption of cilia may alter the morphology and function of other parts of the neuron, and thus, the manipulation might cause some indirect effects on neuron morphology which may alter connectivity or excitability. Based on our findings and the discussion above, we propose a model in which cilia in the striatum act as a calibrator of the timing function of the basal ganglia-cortical circuit by maintaining proper timing perception. According to this model, abnormal cilia functions might be a unifying factor contributing to the pathophysiology of neurological and psychiatric disorders, particularly as related to the deficits in timing judgment.

In conclusion, our findings shed light on the roles that striatal cilia may play in timing-dependent functions. These findings enhance our understanding of brain function in the context of the crucial roles played by this previously unappreciated organelle and may open new avenues for therapeutic intervention through cilia-targeted therapies.

## Supplementary Information

Below is the link to the electronic supplementary material.

Supplementary file1 (DOCX 5791 KB)

## Data Availability

Data that support the findings of this study are available from the corresponding author upon request.

## Declarations

### Research Resource Identifiers (RRID)

AAV.CamKII.HI.GFP-Cre.WPRE.SV40 was a gift from James M. Wilson (Addgene viral prep # 105551-AAV9 http://n2t.net/addgene:105551; RRID:Addgene_105551).

AlexaFluor546 goat anti-rabbit: A101035 Lot: 2273730, Invitrogen, USA.

Anti-c-Fos antibody: ab190289 Rb pAb to c-Fos Lot#: GR339395, Abcam, USA.

Anti-ADCY3 antibody: LSBIO-C204505 ADCY3 Lot#:193037, LSBIO, USA.

DAPI, 1:10,000, Thermo Scientific Ref: 62248 Lot: WF3296471.
