# Supplementary material for: Cilia in the Striatum Mediate Timing-Dependent Functions
Source: Mol Neurobiol. 2022 Nov 2;60(2):545–65. doi: 10.1007/s12035-022-03095-9 (PMC9849326; doi:10.1007/s12035-022-03095-9)
Supplement: Supplementary file 1 — Supplementary file1 (DOCX 5791 KB) [file 12035_2022_3095_MOESM1_ESM.docx]

**Supplemental information**

**Cilia in the Striatum Mediate Timing-Dependent Functions**

Wedad Alhassen^1^, Sammy Alhassen^1^, Jiaqi Chen^1^, Roudabeh Vakil Monfared^1^, Amal Alachkar^1,2,3*^

1 Department of Pharmaceutical Sciences, School of Pharmacy, University of California-Irvine, CA 92697, USA.

2 UC Irvine Center for the Neurobiology of Learning and Memory, University of California-Irvine, Irvine, CA 92697, USA.

3 Institute for Genomics and Bioinformatics, School of Information and Computer Sciences, University of California-Irvine, CA 92697, USA.

*** Corresponding Author**:

Amal Alachkar

Department of Pharmaceutical Sciences

University of California, Irvine

356A Med Surge II

Irvine CA, 92697-4625

Phone; 949-824-2522

[aalachka@uci.edu](mailto:aalachka@uci.edu)

**Keywords:** Cilia, Striatum, Timing, Behaviors

**Supplemental Figures**

- **Figure S1. Effects of cilia removal in the dorsal striatum on cFos expression in the brain structures**
- (a) Representative images of cFos immunostaining in brain structures
- (b) Quantification of the cFos positive cells in the control and IFT88-KO mice. Two way ANOVA, control vs IFT88-KO (F(1, 143) = 50.72, *P*<0.0001), **P*<0.05, ***P*<0.01, *****P*<0.0001, ns, not significant. Data are presented as means±S.E.M; n=3 sections of 5 mice per group.

a

a

b
